# Ultrasound of the Small Bowel in Crohn's Disease

**DOI:** 10.1155/2012/964720

**Published:** 2012-02-21

**Authors:** Emma Calabrese, Francesca Zorzi, Francesco Pallone

**Affiliations:** Cattedra di Gastroenterologia, Dipartimento di Medicina, Università di Roma “Tor Vergata”, 00133 Rome, Italy

## Abstract

Several radiological and endoscopic techniques are now available for the study of inflammatory bowel diseases. In everyday practice, the choice of the technique to be used depends upon its availability and a careful evaluation of diagnostic accuracy, clinical usefulness, safety, and cost. The recent development of innovative and noninvasive imaging techniques has led to a new and exciting area in the exploration of the gastrointestinal tract, especially in Crohn's disease patients by using ultrasound with oral or intravenous contrast.

## 1. Introduction

The diagnosis of Crohn's disease (CD) is based on clinical, endoscopic, radiological, and histological criteria. The main innovations in diagnostic technologies include the development of more sophisticated endoscopic and noninvasive imaging techniques with the aim of improving the identification of complications. Noninvasive tests for the diagnosis and followup of CD have gained increasing attention. Rapid and inexpensive noninvasive tests that are sensitive, specific, and simple to perform are necessary to prevent patient discomfort, delay in diagnosis, and unnecessary costs.

The use of transabdominal ultrasound (US) to evaluate gastrointestinal (GI) tract disorders is used primarily in the assessment of acute and chronic inflammatory conditions such as appendicitis, diverticulitis, ulcerative colitis, and CD [1]. Over the past few years, the technical evolution of ultrasound equipment, combined with the use of oral and intravenous contrast agents and the increased expertise of the operators, has led to a great enthusiasm for ultrasound assessment of the GI tract [2]. In chronic inflammatory conditions, mainly CD, these properties have not only been employed for diagnostic purposes but also been proposed for management and followup of the disease and its complications [3–7]. 

Bowel US has been largely promoted in continental Europe, where ultrasonography is carried out by a physician and is an integral part of the training curriculum for internal medicine, gastroenterology, surgery, and other fields. This technique is available in most European centers due to this training curriculum, whereas its use is less widespread in the United States.

## 2. CD Diagnosis

Bowel U.S. is now becoming the first-line imaging procedure in patients with suspected CD for early diagnosis of the disease [8]. Several studies have evaluated the significance of the U.S. detection of bowel wall thickness in the diagnosis of CD. Prospective studies, performed in unselected groups of patients, have shown that bowel U.S. may diagnose CD with a sensitivity ranging from 67–96% and specificity ranging from 79–100% [9–18]. Most of the results were obtained from studies that included patients with a previous diagnosis of CD but lacked of a control population; thus, it is difficult to achieve a comprehensive evaluation of sensitivity and specificity of the U.S. technique. These methodological problems were evaluated by Fraquelli and Conte [19]. In their meta-analysis, in which only five case-control and two cohort studies were ultimately considered from an initial 44 full-text studies identified, the impact of different cut-off values of bowel wall thickening (3 mm versus 4 mm) in determining the presence of CD was evaluated. The authors concluded that, using a cutoff level of 3 mm as normal, sensitivity and specificity were 88% and 93%, respectively. In contrast, when a cutoff level of ≥4 mm was used, the sensitivity was 75% and specificity 97%. The meta-analysis conducted by Horsthuis et al. evaluated the relevance of US in the detection of IBD in comparison with other techniques [20]. No significant differences in diagnostic accuracy among the imaging techniques were observed. The authors concluded that because patients with IBD often needed frequent reevaluation of disease status, use of a diagnostic modality that does not involve the use of ionizing radiation is preferable [20]. However, the results also show that bowel US may, even in expert hands, be compounded by false-positive and false-negative findings. Thickening of the bowel walls is not specific for CD, also being present in infectious, neoplastic, and other inflammatory diseases [21]. Bowel US may also provide false-negative results, even in the hands of experienced ultrasonographers, for example, in obese patients or those with anorectal lesions only, or when the bowel disease is characterized by only superficial lesions, such as rare aphthous ulcers or mucosal erosions [22]. Interobserver agreement between sonographers with variable experience in bowel ultrasound has been reported in a few preliminary studies showing satisfactory results, but a learning curve for this technique is still lacking [14, 23]. This is probably one of the main reasons why bowel US is, in clinical practice, still regarded with skepticism by many clinicians and radiologists [6, 23]. 

**Figure 1:**
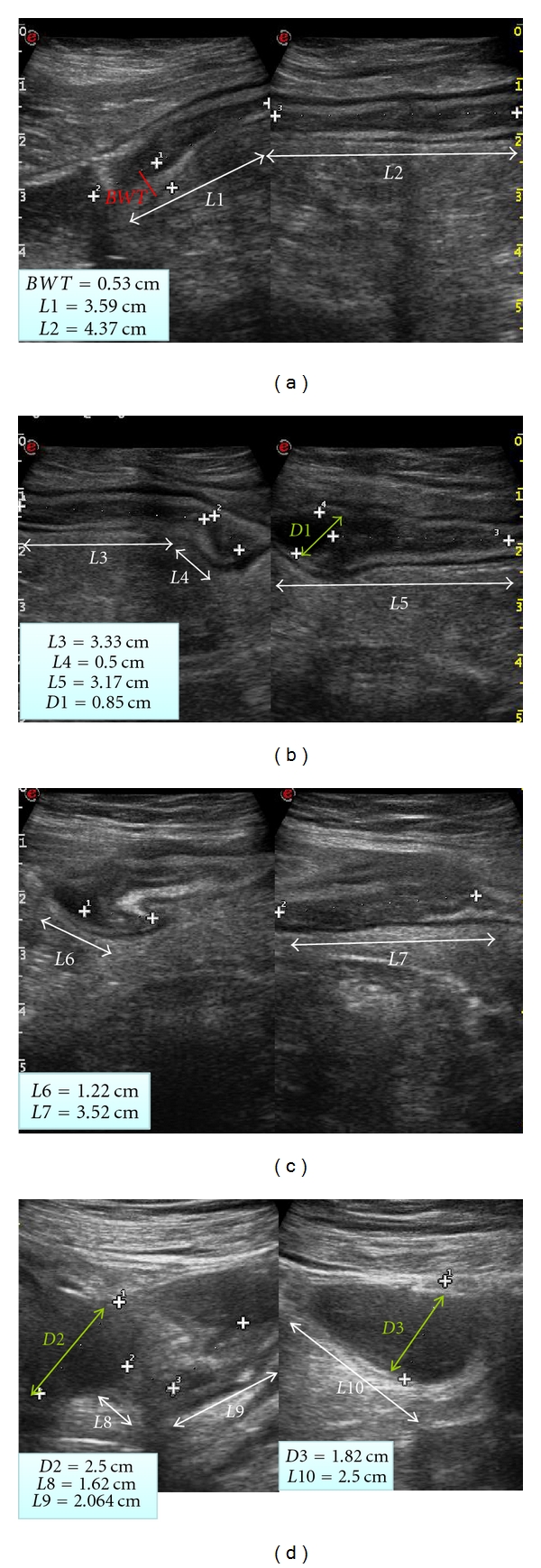
Small intestine contrast ultrasonography in a 20-year-old female with Crohn's disease. In each panel ((a)–(d)) white arrows indicate disease extent of the terminal ileum. The cumulative extent of the sonographic Crohn's disease lesion was 26 cm. In (a), on the left side, red arrow indicates bowel wall thickness (5.3 mm) of the terminal ileum and in (b) (on the left side) and (d) green (both right and left sides) arrows indicate lumen diameter (raging from 8 to 22 mm) at level of the terminal ileum.

The use of oral contrast agents such as iso-osmolar polyethylene glycol solution (PEG; at a volume ranging from 375–800 mL) during ultrasound assessment has been proposed to define CD lesions with improved accuracy [24–26] (Figure 1). Because of the small amount of fluid ingested (usually no more than 500 mL) and its palatability, this procedure has been reported to be wellaccepted and safe. None of the studies have reported significant side effects or major complaints during or immediately after PEG ingestion. The use of PEG appears to reduce intraobserver variability between sonographers and to increase sensitivity in defining disease extent, lesion site, and bowel complications of CD; thus, it has value in the early diagnosis and in the followup of CD [26, 27]. These findings suggest that small intestine contrast ultrasonography (SICUS) may be used as an alternative technique to invasive procedures to assess ileal lesions and monitor their progression over time. 

## 3. Stenosis

**Figure 2:**
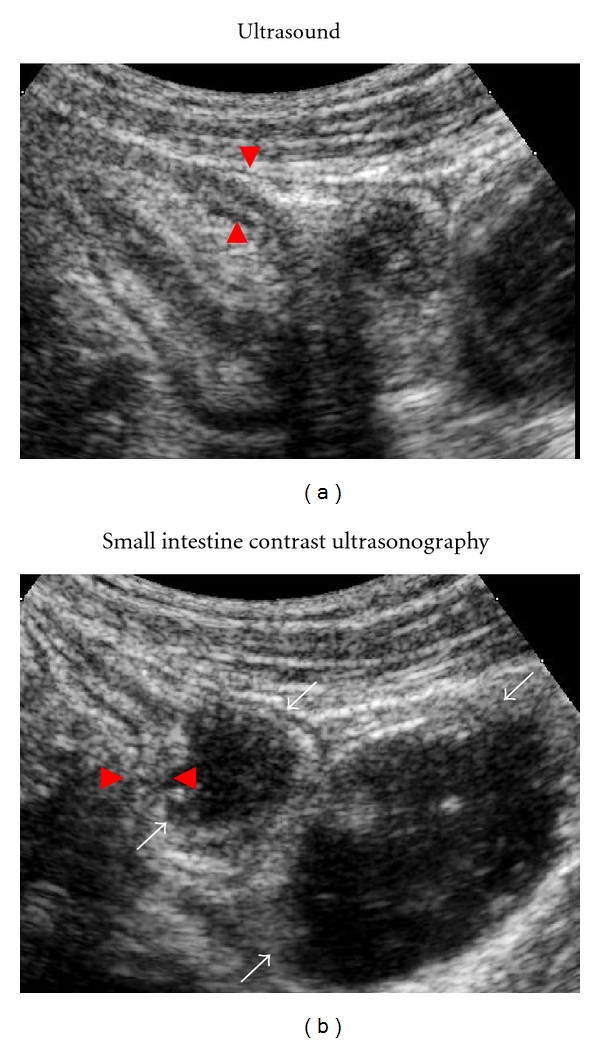
A 30-year-old male with stricturing Crohn's disease assessed by bowel ultrasound (without oral contrast) (a) and small intestine contrast ultrasonography (b). (a) shows Crohn's disease stenosis and prestenotic dilation, well defined in (b). Red arrowheads indicate bowel wall thickness in both panels, white arrows indicate prestenotic dilation in (b).

Bowel US currently detects stenosis in 70–79% of unselected CD patients and in >90% of those with severe bowel stenoses needing surgery, with false-positive diagnoses limited to 7% [3, 4, 27, 28]. The use of PEG leads to a significantly greater accuracy of bowel ultrasound in detecting the presence and the number of stenoses. Bowel US with oral contrast detected at least one stenosis and at least two stenoses in >10% and >20% more patients, respectively, in comparison with bowel US without oral contrast agents, resulting in a sensitivity of approximately 90% for detection of a single stenosis and >75% for detection of multiple stenoses (Figures 2(a) and 2(b)) [26, 27]. 

## 4. Fistula

**Figure 3:**
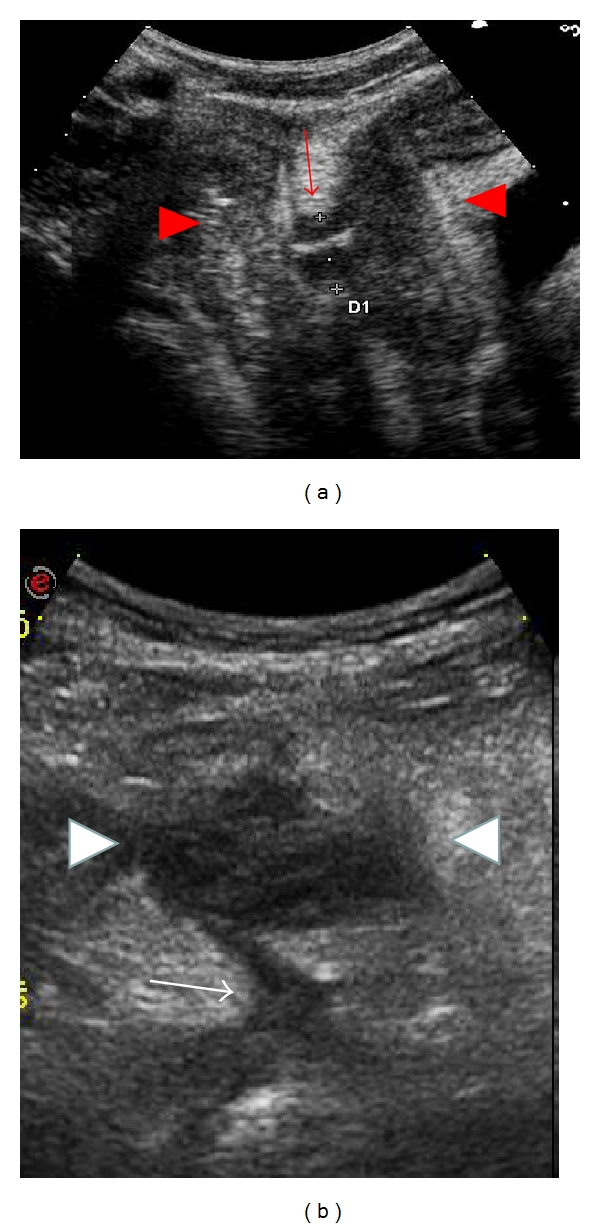
Bowel ultrasound in a 22-year-old female with ileocolonic Crohn's disease. In (a) red arrow indicates enteroenteric fistula (defined as a hypoechoic track with a hyperechoic content) between diseased ileal loops with bowel wall thickness (red arrowheads). In (b) a small abscess (white arrowheads identify a roundish anechoic lesions with irregular walls, presenting internal echoes) with fistula (white arrow) was identified in the same patient.

Two prospective studies have evaluated the role of US (without oral contrast) in determining the presence of internal fistulae (Figure 3(a)) using surgical and surgical-pathological findings as the reference standard. In one study, Gasche et al. reported a sensitivity of 87% and a specificity of 90% for bowel US in the detection of internal fistulae [4]. In the other prospective study, Maconi et al. determined the accuracy of bowel US and X-ray studies for detecting internal fistulae to be comparable with a sensitivity of 71.4% for US and 69.6% for X-ray, and specificity of 95.8% for both techniques. Maconi et al. showed also that the combination of these two techniques significantly improved preoperative diagnostic performance (sensitivity 97.4% and specificity 90%), with US being more accurate in detecting enteromesenteric fistulae while X-ray studies were superior in the diagnosis of enteroenteric fistulae [29]. In a recent study by Pallotta et al., SICUS identified fistulae in 27/28 patients and excluded it in 19/21 patients (96% sensitivity, 90.5% specificity) using surgery as gold standard [30]. In a recent systematic review, Panes and colleagues evaluated diagnostic accuracy of cross-sectional imaging techniques (US, CT and MR) for diagnosis of fistulas. These techniques showed higher accuracy than that of small bowel follow through (SBFT) [31]. CT and MR enterography showed similar accuracy for the identification of extraenteric complications (sensitivity for both) [32, 33].

## 5. Abscess

Computed tomography (CT) and magnetic resonance imaging (MRI) are considered to be nonsurgical gold standard for the diagnosis of CD-related abscesses [31]. However, bowel US is also considered as a first-level procedure mainly because it is simple to use (Figure 3(b)). Four studies have prospectively assessed the accuracy of bowel US in the detection of intraabdominal abscesses, showing a mean sensitivity and specificity of 91.5% and 93%, respectively [3, 4, 29, 34]. In these studies, US showed a higher sensitivity in the detection of superficial intraperitoneal abscesses, whereas the diagnosis of deep pelvic or retroperitoneal abscesses was more difficult due to the presence of overlying bowel gas. Pallotta et al. showed that intraabdominal abscesses were correctly detected in 10/10 patients and excluded in 37/39 patients (100% sensitivity, 95% specificity, *k* = 0.89) by SICUS [30]. Contrast-enhanced ultrasound (CEUS) can be used to distinguish abscesses from inflammatory infiltrates [35]. 

## 6. Postoperative Recurrence

The sensitivity of bowel US in identifying the endoscopic recurrence after ileocolonic resection has been investigated in two studies showing 82% sensitivity [6, 36]. The use of PEG solution increased the sensitivity of ultrasound for assessing CD recurrence in patients under regular followup after ileocolonic resection. In our series, SICUS showed a high sensitivity (92.5%), positive predictive value (94%), and accuracy (87.5%) for detecting CD recurrence lesions using ileocolonoscopy as the gold standard [7].

## 7. CD Activity

The role of bowel US in the assessment of CD activity remains controversial. The degree of bowel wall thickening and extent of the thickened bowel wall on US (as an index of activity in CD) showed a significant but weak direct correlation between these features and clinical and biochemical parameters [22]. However, a statistically significant correlation was found between maximum bowel wall thickness and disease activity score in children and young adults [37]. 

Several studies have focused on the vascularity within the diseased bowel walls, assessed by power-Doppler US, as a quantitative method for determining CD activity. Three studies used Doppler US for detection of active disease, showing that wall thickness and vascularization pattern are useful for detection of active disease [38–40]. A recent study evaluating flow of the superior mesenteric artery confirmed previous observations regarding the correlation between disease activity and Doppler parameters [41–43]. 

The effectiveness of intravenous contrast agents in detection and assessing of bowel US activity of CD, despite some positive findings, remains controversial [40, 44–47]. The introduction of the microbubble contrast agents has enabled US to obtain information regarding the perfusion behavior of the organs and their diffuse or focal diseases. Enhancement in different wall layers can be evaluated and quantified in CD and correlates to clinical activity indices [35]. Migaleddu et al. reported in a prospective study that contrast-enhanced ultrasound (CEUS) showed 93.5% sensitivity, 93.7% specificity, and 93.6 overall accuracy in detecting inflammatory activity, calculated using the endoscopy/biopsy as gold standard. The linear correlation coefficient for CEUS versus Crohn's disease activity index (CDAI) was 0.74 (*P* < 0.0001) [40]. Ripolles et al. reported in a prospective study sensitivity and specificity of 96% and 73%, respectively, in the prediction of moderate or severe grade for inflammation at CEUS using endoscopy as gold standard [45]. More studies are needed to establish the exact role of CEUS in the imaging of GI pathology [35].

## 8. Conclusion 

In recent years, several radiological and endoscopic techniques have been developed for the study of the small bowel. Bowel US has now become the first-line imaging procedure in patients with suspected CD for its early diagnosis. However, since the procedure is easy to use and offers good repeatability and accuracy, the most important indication of bowel US is currently in the followup of patients known to have CD. CEUS has been introduced as effective method in the quantitative and qualitative evaluation of CD inflammatory activity. In this context, these techniques may play a pivotal role in the early detection of intraabdominal complications, such as strictures, fistulae, and abscesses, and may be useful in the assessment of activity and in monitoring the course of disease during medical and postoperative followup, as a prognostic index of recurrence.
